# EOESGC: predicting miRNA-disease associations based on embedding of embedding and simplified graph convolutional network

**DOI:** 10.1186/s12911-021-01671-y

**Published:** 2021-11-16

**Authors:** Shanchen Pang, Yu Zhuang, Xinzeng Wang, Fuyu Wang, Sibo Qiao

**Affiliations:** 1 College of Computer Science and Technology, China University of Petroleum, Qingdao, China; 2 College of Mathematics and Systems Science, Shandong University of Science and Technology, Qingdao, China

**Keywords:** miRNA-disease associations, Embedding of embedding, Simplified graph convolutional network, Coupled heterogeneous graph

## Abstract

**Background:**

A large number of biological studies have shown that miRNAs are inextricably linked to many complex diseases. Studying the miRNA-disease associations could provide us a root cause understanding of the underlying pathogenesis in which promotes the progress of drug development. However, traditional biological experiments are very time-consuming and costly. Therefore, we come up with an efficient models to solve this challenge.

**Results:**

In this work, we propose a deep learning model called EOESGC to predict potential miRNA-disease associations based on embedding of embedding and simplified convolutional network. Firstly, integrated disease similarity, integrated miRNA similarity, and miRNA-disease association network are used to construct a coupled heterogeneous graph, and the edges with low similarity are removed to simplify the graph structure and ensure the effectiveness of edges. Secondly, the Embedding of embedding model (EOE) is used to learn edge information in the coupled heterogeneous graph. The training rule of the model is that the associated nodes are close to each other and the unassociated nodes are far away from each other. Based on this rule, edge information learned is added into node embedding as supplementary information to enrich node information. Then, node embedding of EOE model training as a new feature of miRNA and disease, and information aggregation is performed by simplified graph convolution model, in which each level of convolution can aggregate multi-hop neighbor information. In this step, we only use the miRNA-disease association network to further simplify the graph structure, thus reducing the computational complexity. Finally, feature embeddings of both miRNA and disease are spliced into the MLP for prediction. On the EOESGC evaluation part, the AUC, AUPR, and F1-score of our model are 0.9658, 0.8543 and 0.8644 by 5-fold cross-validation respectively. Compared with the latest published models, our model shows better results. In addition, we predict the top 20 potential miRNAs for breast cancer and lung cancer, most of which are validated in the dbDEMC and HMDD3.2 databases.

**Conclusion:**

The comprehensive experimental results show that EOESGC can effectively identify the potential miRNA-disease associations.

## Background

As a kind of non-coding RNA (ncRNA), miRNA was once thought to be the medium of transcriptional noise from RNA to protein [1–4]. However, this idea was proved wrong, and it was verified that non-coding RNA plays an important role in various biological effects [1, 5]. MiRNA is endogenous, evolutionarily conserved single stranded ncRNA that regulates gene expression through complementary base pairing with corresponding target RNA (mRNA) sequences [6–8]. More and more studies had shown that miRNA was closely related to the generation of complex diseases, such as various cancers, diabetes, Alzheimer’s disease and other diseases [9–13]. In particular, miRNA act as oncogenes or tumor inhibitors in the generation and metastasis of some cancers, including breast cancer [11] and lung cancer [13]. An important goal of medical data modeling and classification is to make predictions based on training data and available features. Medical data sets with high dimensional feature space and relatively small sample numbers are key problems in machine learning tasks [14]. Therefore, more and more researchers hope to use intelligent models to predict the potential association between miRNA and disease based on the existing proven data of miRNA and disease. Most of the methods proposed so far rely on the hypothesis that functional similarity of miRNAs is associated with similar diseases [15]. The following are several methods for predicting miRNA-disease associations based on graph encoders, random walk, machine learning, and graph convolutional neural network.

Nowdays, graph neural networks have shown their superior performance, such as graph autoencoder. Ji et al. [16] proposed a semi-supervised model (SVAEMDA), which was a novel feature learning approach to obtain their feature representations from an integrated set of miRNA and disease similarity networks. SVAEMDA used known miRNA-disease associations in the form of cascaded dense vectors to train predictors based on variable auto-encoders. The reconstruction probability of predictors was used to measure the micronucleic miRNA-disease associations. In addition, the model did not need to use negative samples to reduce noise data. Zhang et al. [17] also proposed an unsupervised deep learning framework with variable autoencoder to predict miRNA-disease associations by constructing two spliced matrices as autoencoder (VAE) inputs where VAE learned the potential representation of input and reconstructed the data from the learned distribution. The association score of miRNA-disease was obtained by using the trained VAE model. Liu et al. [18] proposed a framework based on stacked autoencoder and XGBoost to predict the potential miRNA-disease associations (SMALF). This model differs from the two previous models as it used an autoencoder to extract miRNA and potential feature vectors of disease, rather than acting as a classifier. It used XGBoost to predict positional miRNA-disease associations. Ding et al. [19] proposed a new computational model based on variational graph auto-encoder with matrix factorization (VGAMF) for miRNA-disease associations prediction. The innovation of this model is to use two autoencoders to obtain miRNA and disease feature representation on miRNA similarity network and disease similarity network respectively. This is something that no other model has used.

Secondly, motivated by word2vec, a random walk algorithm was used in the graph to obtain the sequence of nodes and thus the embedding representation of the nodes. Numerous studies had confirmed that the use of a random walk algorithm can effectively predict miRNA-disease associations. Niu et al. [20] constructed a prediction model based on the random walk and binary regression, which extracted the features of the miRNAs by restarting the random walk and used binary logistic regression to score the new miRNA-disease associations. Li et al. [21] proposed a three-layer heterogeneous network combined with a non-equilibrium random walk for the miRNA-disease associations’ prediction model (TCRWMDA). This model enabled the construction of a three-layer heterogeneous network, which enriched the information in the basic network and enabled the mining of more effective information between the networks. Dai et al. [22] proposed a double random walk based on a Logistic weighted profile to explore the miRNA-disease associations model (LWBRW). The special feature during the process of constructing this network. A logistic function was used to extract valuable information. Weighted known proximity (WKNKN) was used to preprocess the known association matrix, and the new miRNA-disease associations were inferred by double random walk on the miRNA network and the disease network using the LWBRW method.

Thirdly, traditional machine learning methods are simple but still have good results. The random forest algorithm had also made outstanding contributions in miRNA-disease associations prediction. Chen et al. [23] proposed a random forest-based method to predict the miRNA-disease associations (RFMDA), using feature selection based on positive and negative sample feature frequencies to reduce the dimension of the sample space. A random forest model was trained to obtain an association score between miRNA and disease. Later, Yao et al. [24] proposed an improved RF model (IRFMDA). Different from Chen’s multi-attribute decision analysis method, this model utilized the importance score of RF variables to realize feature selection, which could effectively reduce the influence of redundancy and noise information, and selected more valuable samples to represent samples, thus improving the prediction ability of the model. Zheng et al. [25] proposed a machine learning approach (MLMDA) to predict and verify miRNA-disease associations by integrating heterogeneous information sources. This model used the k-mer sparse matrix to extract miRNA sequence information and other similarity information, which then implements an autoencoder to extract the most representative features of these features. In the end, random forest classifiers are deployed to predict miRNA-disease associations. Chen et al. [26] proposed a novel rank-based KNN-based miRNA-disease associations prediction calculation method (RKNNMDA) to predict potential miRNA-disease associations. K-nearest neighbor (KNN) algorithm was used to search for miRNA and disease. Then the k-nearest neighbors were reordered according to the SVM sorting model. Finally, a weighted vote was conducted to obtain a final ranking of all possible miRNA disease associations.

Finally, graph convolutional neural networks have shown powerful advantages in the processing of complex graphs, which has led to an increasing number of researchers using graph convolutional neural networks to solve problems. Peng et al. [27] implemented a convolutional neural network-based framework (MDA-CNN) for predicting miRNA-disease associations by combining similarities between miRNA, similarities between diseases, and interactions between proteins. Chu et al. [28] proposed a new graph sampling method by using feature graph and topology graph to identify miRNA-disease associations (MDA-GCNFTG) through graph convolution. This method was modeled based on the potential associations of feature space and the structural relationship of miRNA-disease associations data where this model could predict not only new miRNA-disease associations but also new disease-related miRNAs under unbalanced sample distribution. Tang et al. [29] proposed a multi-view and multi-channel attention convolutional network to predict the potential miRNA-disease associations (MMGCN). GCN was used to extract miRNA and node features from different similarity views, and the model used node embedding learned from multi-channel attentional enhancement to make association predictions. Li et al. [30] proposed a neural inductive matrix completion with a graph convolutional network (NIMCGCN) approach to predict miRNA disease association. First, a graph convolutional network (GCN) was used to learn miRNA and disease underlying feature representation. Then, the learned features were input into a new neural induced completion matrix (NIMC) model to generate the completion correlation matrix. The approach used supervised end-to-end learning to effectively predict miRNA-disease associations.

In conclusion, most of the miRNA-disease associations’ prediction frameworks have been proposed using the embedding of a single model learning node. Both of them ignore the edge information of the Coupled heterogeneous graph, the edge between networks can act as supplementary information of nodes. This supplementary information is important because it makes potential feature more complete and accurate. The framework we have proposed is to fill that gap. We use the EOE model based on the link to learn edge features and add them into node embedding as supplementary information. The SGC model is used for information aggregation. By combining the two models, learning edge information and aggregating neighbor information enables each node embedding to contain richer information, which also lays the foundation for effective prediction of miRNA-disease potential associations.

## Methods

**Fig. 1:**
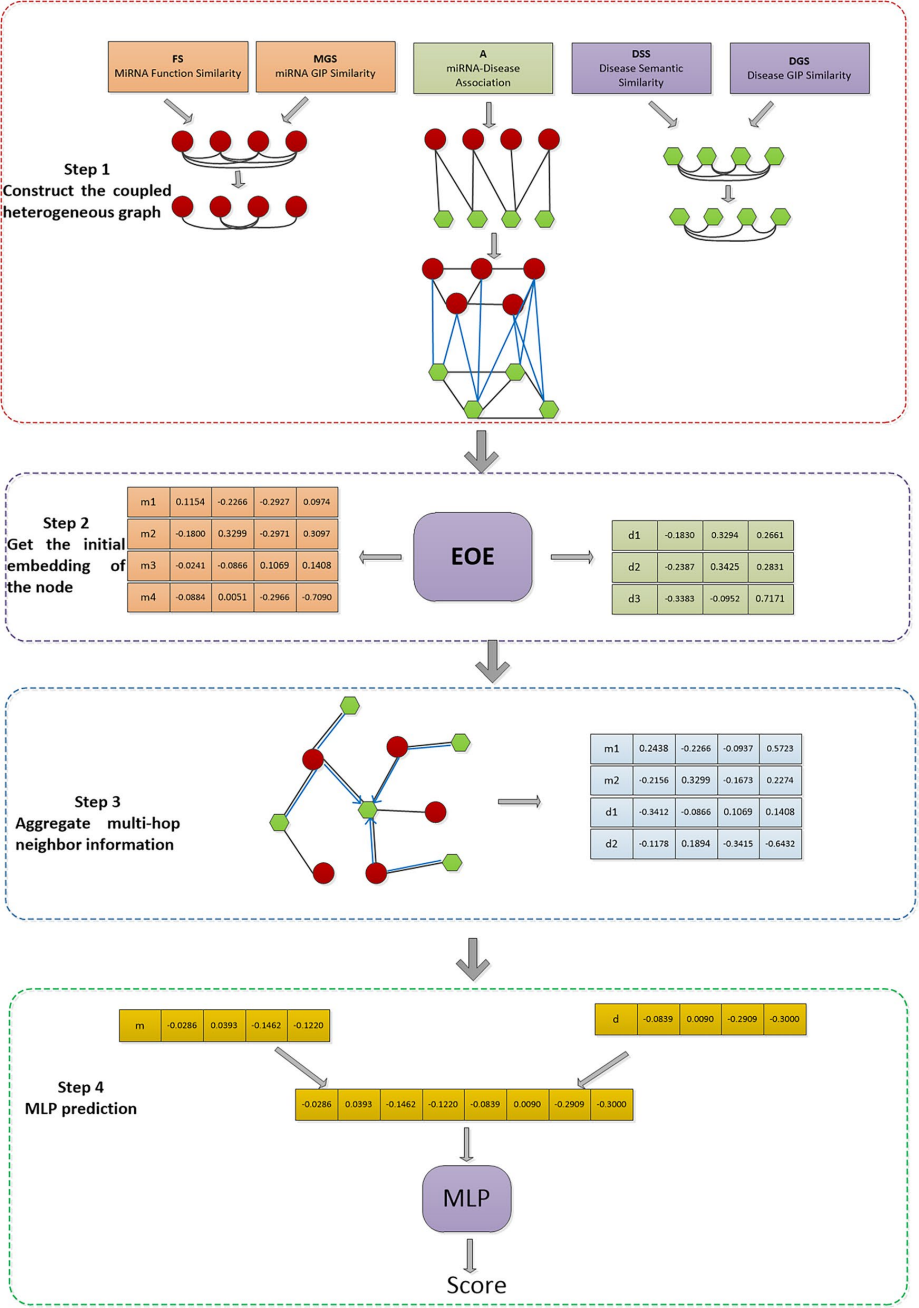
Flow chart of EOESGC. Step 1 is to construct the coupled heterogeneous graph. FS is the functional similarity of miRNA, MFS is the Gaussian kernel similarity of miRNA, DSS is the semantic similarity of disease, DGS is the Gaussian kernel similarity of disease, and A is miRNA-disease association matrix. Step 2 is to use the EOE model to learn edge information. Step 3 uses the SGC model to aggregate node information. Step 4 uses MLP to predict miRNA-disease association score

We present a novel framework for predicting the potential miRNA-disease associations. As shown in Fig. 1, the framework consists of four steps in total:

- The first step is to construct the coupled heterogeneous graph, where we use the disease similarity, miRNA similarity, and confirmed miRNA-disease association networks to construct the graph and remove the edges with less similarity to reduce the complexity of the graph.
- The second step is using the link-based node embedding model-EOE to add network edge information to node features.
- The third step is to use the SGC model for feature aggregation to fully learn the structural information of the graph, and finally get the low dimensional embedding of the node.
- The last step is to feed the final embedding splicing into the MLP for prediction.

### Database

A coupled heterogeneous graph consists of two distinct but related sub-nets connected by inter-network edges [31]. Consists of two distinct but related sub-nets connected by inter-network edges. The term “different” implies that the vertices of the two sub-networks are of different node types. The term “correlation” implies that the vertices of two sub-networks have a particular interaction. To construct a miRNA-disease coupled heterogeneity graph, we downloaded data from the HMDD2.0 database [32] containing 495 miRNAs, 383 diseases, and 5430 confirmed miRNA-disease associations. We use the adjacency matrix A to represent miRNA-disease associations where $A_{ij}=1$ means there is an interaction between miRNA i and disease j, while $A_{ij}=0$ means there is no relationship. In the experiment stage, we used dbDEMC [33] and HMDD3.2 databases as the verification database to verify the accuracy of the EOESGC model we proposed.

### Disease similarity network

We effectively combine disease semantic similarity with a disease Gaussian interaction profile kernel similarity to construct disease similarity network. To ensures edges among disease nodes are valid, we set a threshold and remove the link below the threshold. Therefore, the disease similarity is calculated as follows:

1 $$\begin{aligned} DS^{\prime }(d_{i},d_{j})=\alpha \frac{DSS^{1}(d_{i},d_{j})+DSS^{2}(d_{i},d_{j})}{2}+(1-\alpha )DGS(d_{i},d_{j}) \end{aligned}$$

The first semantic similarity is $DSS^{1}$, the second semantic similarity is $DSS^{2}$, and the Gaussian interaction profile kernel similarity is *DGS*. In the experiment, $\alpha$ represents a scaling factor. The disease similarity obtained after removing data with low similarity according to the threshold h:

2 $$\begin{aligned} DS(d_{i},d_{j})= {\left\{ \begin{array}{ll} DS^{\prime }(d_{i},d_{j})&{}\quad {DS^{\prime }(d_{i},d_{j}) \ge h}\\ 0&{}\quad {\text{other else}} \end{array}\right. } \end{aligned}$$

#### Disease semantic similarity model 1

**Fig. 2:**
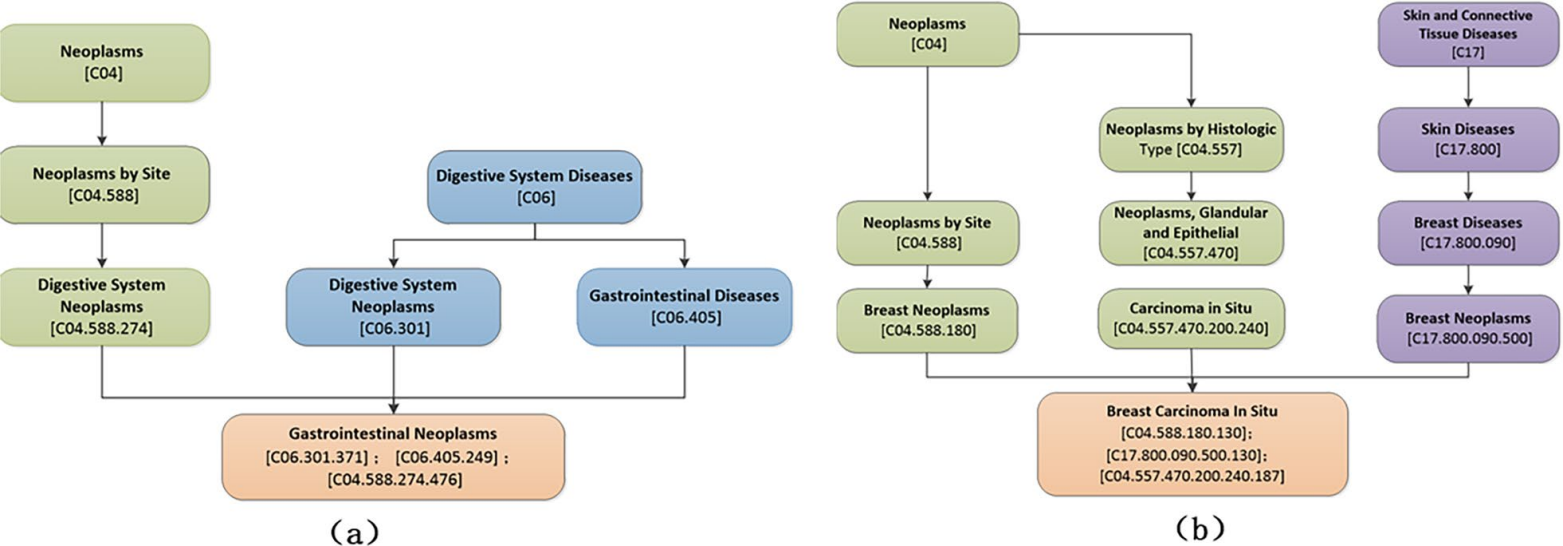
Directed acyclic graph of diseases. **a** Directed acyclic graph of breast cancer in situ, **b** directed acyclic graph of gastrointestinal neoplasms

Medical subject headings (MESH) [34] is the authoritative subject list compiled by the United States National Library of Medicine. It is a normalized and expandable dynamic thesaurus. Mesh is a collection of more than 18,000 medical topics that we use to study the relationships between diseases. The disease can be described as a directed acyclic graph (DAG = $N_{d}, E_{d}$), where $N_{d}$ is the node-set of d and it’s ancestor nodes, $E_{d}$ is edge set [35]. Figure 2 shows the DAG of two diseases.

To calculate the similarity of two disease semantics based on DAG(D), we need to calculate the semantic contribution score for each disease in the graph. We define the contribution score of disease d to disease D in DAG(D) as:

3 $$\begin{aligned} D^{1}_{D}= {\left\{ \begin{array}{ll} 1&{} \quad {d = D}\\ max \left\{ \Delta *D^{1}_{D}(d^{\prime })|d^{\prime } \in the \; children \; of \; d \right\} &{}\quad {d\ne D} \end{array}\right. } \end{aligned}$$

where $\Delta =0.5$ is a decay factor indicating that the more distant nodes from disease D contribute less to the semantics of disease D. The semantic value of disease D is calculated based on the semantic contribution score of the disease nodes in DAG(D).

4 $$\begin{aligned} DV^{1}(D)=\sum _{d \in N(D)}D^{1}_{D}(d) \end{aligned}$$

If DAG (A) and DAG (B) have same diseases, we consider disease A and disease B to be similar. Therefore, the first semantic similarity between two diseases is defined as:

5 $$\begin{aligned} DSS^{1}=\frac{\sum _{t \in N(d_{i})\cap N(d_{j})}\big (D^{1}_{d_{i}}(t)+D^{1}_{d_{j}}(t)\big )}{DV^{1}(d_{i})+DV^{1}(d_{j})} \end{aligned}$$

#### Disease semantic similarity model 2

Xuan et al. [36] defined the essential difference between the second disease semantic similarity and the first disease semantic similarity which differs in the calculation of the semantic contribution of disease nodes. The ancestor nodes of disease D have d1 and d2, and if d1 appears less frequently in DAG than d2, then we believe that d1 has a greater semantic contribution to disease D. Therefore, the semantic contribution score of disease node d to disease D is defined as:

6 $$\begin{aligned} D^{2}_{D}(d)=-log\left( \frac{{\text{the number of }}DAG_{s}{\text{ including d}}}{{\text{the number of disease}}}\right) \end{aligned}$$

As in model 1, the semantic value of each disease and the semantic similarity of the two diseases are defined as:

7 $$\begin{aligned} DV^{2}(D)= & {} \sum _{d \in N(D)}D^{2}_{D}(d) \end{aligned}$$

8 $$\begin{aligned} DSS^{2}= & {} \frac{\sum _{t \in N(d_{i})\cap N(d_{j})}\big (D^{2}_{d_{i}}(t)+D^{2}_{d_{j}}(t)\big )}{DV^{2}(d_{i})+DV^{2}(d_{j})} \end{aligned}$$

#### Disease Gaussian interaction profile kernel similarity

Since not all the diseases can be found in the MESH, we use the disease Gaussian interaction profile kernel similarity (GIP) as a supplement. GIP similarity is calculated for miRNA and disease respectively using the method proposed by Zhao et al. [37]. The adjacency matrix $A \in R^{m *n}$ of miRNA-disease, where each column is used to represent a disease, is defined as IP(D), where each column is defined as IP(D) to represent a disease. Then, the Gaussian interaction kernel similarity between diseases $d_{i}$ and $d_{j}$ is defined as:

9 $$\begin{aligned} KD(d_{i},d_{j})=exp\big (-\gamma _{d} ||IP(d_{i})-IP(d_{j})||^{2}\big ) \end{aligned}$$

where $\gamma _{d}$ is used to control kernel bandwidth, $\gamma _{d}^{\prime }$ is usually set to 0.5 for controlling the kernel bandwidth $\gamma _{d}$ is defined as:

10 $$\begin{aligned} \gamma _{d}=\gamma _{d}^{\prime }/\frac{1}{n}\sum ^{n}_{i=1}||IP(d_{i})||^{2} \end{aligned}$$

### MiRNA similarity network

We use miRNA functional similarity and Gaussian interaction profile kernel similarity to construct miRNA similarity network. The Gaussian interaction profile kernel similarity is the same as in the previous section. miRNA similarity is defined as:

11 $$\begin{aligned} MS^{\prime }(m_{1},m_{2})=\alpha FS(m_{1},m_{2})+(1-\alpha )MGS(m_{1},m_{2}) \end{aligned}$$

where $\alpha$ is the scale factor, FS is the miRNA function similarity. We set a threshold value of h, in believing there is no association between miRNAs with a similarity less than h. Therefore, the final miRNA similarity network is defined as:

12 $$\begin{aligned} MS(m_{1},m_{2})= {\left\{ \begin{array}{ll} MS^{\prime }(m_{1},m_{2})&{}\quad {MS^{\prime }(m_{1},m_{2}) \ge h}\\ 0&{}\quad {\text{other else}} \end{array}\right. } \end{aligned}$$

According to Wang et al. [35] study, miRNAs with similar functions are often associated with diseases with similar semantics, and the relationship between different diseases can be represented by a directed acyclic graph (DAG) structure. The functional similarity of miRNA is inferred by measuring the similarity of DAG of related diseases. Firstly, the similarity of disease $d_{t}$ to the disease set DT is defined as:

13 $$\begin{aligned} S(d_{t},DT)=max_{1\le i \le k}{S(d_{t},d_{i})} \end{aligned}$$

If the disease set associated with $m_{1}$ is $DT_{1}$ and the disease set associated with $m_{2}$ is $DT_{2}$, then the functional similarity between and is defined as:

14 $$\begin{aligned} MS(m_{1},m_{2})=\frac{\sum _{1 \le i \le m}S(d_{i},DT_{2})+\sum _{1 \le j \le n}S(d_{j},DT_{1})}{m+n} \end{aligned}$$

where $d_{i}$ belongs to $DT_{1}$, $d_{j}$ to $DT_{2}$, m is the number of diseases contained in $DT_{1}$, and n is the number of diseases contained in $DT_{2}$.

### EOESGC model

We combine two embedding models to obtain the embedding of nodes. The first is the link-based graph embedding model-Embedding of Embedding model, which proposed a new graph type called coupled heterogeneous graph, and miRNA-disease network essentially belongs to this type. The EOE model emphasizes that linked vertices should be close to each other and unlinked vertices should be far away from each other. The latter rule is also important. Therefore, the model sets different loss functions to satisfy this rule. A harmony matrix M was proposed to calculate the proximity between different types of nodes. The link-based embedding model can learn edge features of graph well and add them to node features as supplementary information, which is effective and easy to implement. Then, we input the obtained embedding and miRNA-disease association network into the simplified graph convolution network to continue learning node features. The nonlinear GCN [38] is transformed into a simple linear model SGC, which reduces the additional complexity of the GCN by repeatedly eliminating the non-linearity between the GCN layers and folding the resulting function into a linear transformation. This simplified linear SGC model is more efficient on many tasks than GCN and some other GNN networks along with fewer parameters as well. And the embedding model based on convolution can effectively obtain the neighbor information of the node. The EOESGC model does not join the embedding of the two models but puts the embedding obtained from one model into the second model for training. The experiment proves that this method can effectively learn node embedding.

#### Embedding of embedding

The EOE uses proximity to measure whether there are links between nodes. The larger the degree of proximity is the more similar between two same types of nodes will be, and correlations between two different types of nodes will show. We input the similarity matrix of nodes as the original feature. So we define the proximity between two nodes of the same type as follows:

15 $$\begin{aligned} p(d_{i},d_{j})= & {} \frac{1}{1+exp\big (-d^{T}_{i}d_{j}\big )} \end{aligned}$$

16 $$\begin{aligned} p(m_{i},m_{j})= & {} \frac{1}{1+exp\big (-m^{T}_{i}m_{j}\big )} \end{aligned}$$

where $d_{i}$ represents row i of the disease similarity matrix, $d_{j}$ represents row j of the disease similarity matrix, $m_{i}$ represents row i of the miRNA similarity matrix, $m_{j}$ represents row j of the miRNA similarity matrix.

For different types of nodes, the feature matrix $M \in R^{m*n}$ is introduced during the calculation of proximity since their features cannot be directly computed in different feature spaces. Thus the proximity between pairs of nodes of different types is defined as:

17 $$\begin{aligned} p(d_{i},m_{j})=\frac{1}{1+exp\big (-d_{i}^{T}Mm_{j}\big )} \end{aligned}$$

In order to satisfy that bounded nodes with small probability and boundless vertices with large probability should receive greater penalties. The loss function is defined as:

18 $$\begin{aligned} \begin{aligned} loss&=-\Bigg [\sum _{(d_{i},d_{j})\in E_{d}}(W_{d})_{ij}log(p(d_{i},d_{j})) \\&\quad +\sum _{(m_{i},m_{j})\in E_{m}}(W_{m})_{ij}log(p(m_{i},m_{j}))\\&\quad +\sum _{(d_{i},m_{j})\in E_{dm}}(W_{dm})_{ij}log(p(d_{i},m_{j}))\Bigg ]\\&\quad -\Bigg [\sum _{(d_{i},d_{j})\notin E_{d}} log(1-p(d_{i},d_{j}))\\&\quad +\sum _{(m_{i},m_{j})\notin E_{m}}log(1-p(m_{i},m_{j}))\\&\quad +\sum _{(d_{i},m_{j})\notin E_{dm}} log(1-p(d_{i},m_{j}))\Bigg ] \end{aligned} \end{aligned}$$

where $E_{d}$ is the set of edges between diseases, $E_{m}$ is the set of edges between miRNAs, $E_{dm}$ is the edge set between disease and miRNA, $W_{d}$ is the similarity matrix of disease, $W_{m}$ is the similarity matrix of miRNA, and $W_{dm}$ is the weight between disease and miRNA.

#### Simplifying graph convolutional network

In the traditional GCN, each layer can only aggregate the information of directly connected neighbors. while in SGC, we can set the information aggregation of K-hop neighbors at each layer. SGC consists of two parts, a fixed feature extractor and a linear logistic regression classifier. In our proposed framework, only the feature extractor is used to obtain the embedded representation of nodes. Because miRNA and disease embedding learned from the EOE model still belong to two different feature spaces, they are first mapped to the same feature space.

We map diseases and miRNAs into the Z dimensional feature space as follows:

19 $$\begin{aligned} X_{m}&=W^{M}\cdot x_{m} \end{aligned}$$

20 $$\begin{aligned} X_{d}&=W^{D}\cdot x_{d} \end{aligned}$$

where $x_{m}$ and $x_{d}$ are miRNA embedding and disease embedding output by EOE, $W^{M}$, $W^{D} \in R^{Z}$ are the mapping matrices. Then, the feature embedding of the disease and miRNA are fed into the SGC. The convolution operation for each layer is as follows:

21 $$\begin{aligned} \tilde{A}= & {} A+I \end{aligned}$$

22 $$\begin{aligned} S= & {} \tilde{D}^{-\frac{1}{2}}\tilde{A}\tilde{D}^{-\frac{1}{2}} \end{aligned}$$

23 $$\begin{aligned} S^{k}= & {} S\cdots SS \end{aligned}$$

24 $$\begin{aligned} \bar{X}= & {} S^{k}X \end{aligned}$$

where A is the adjacency matrix of the graph, I is the identity matrix; D is the degree matrix of A, K is the step size.

Finally, the output disease embedding and miRNA embedding are spliced, and make predictions with MLP. This step uses the cross-entropy loss function to optimize the model.

25 $$\begin{aligned} loss=-[ylog\tilde{y}+(1-y)log(1-\tilde{y})] \end{aligned}$$

where y is the edge label, $\tilde{y}$ is the predicted score.

## Results

**Fig. 3:**
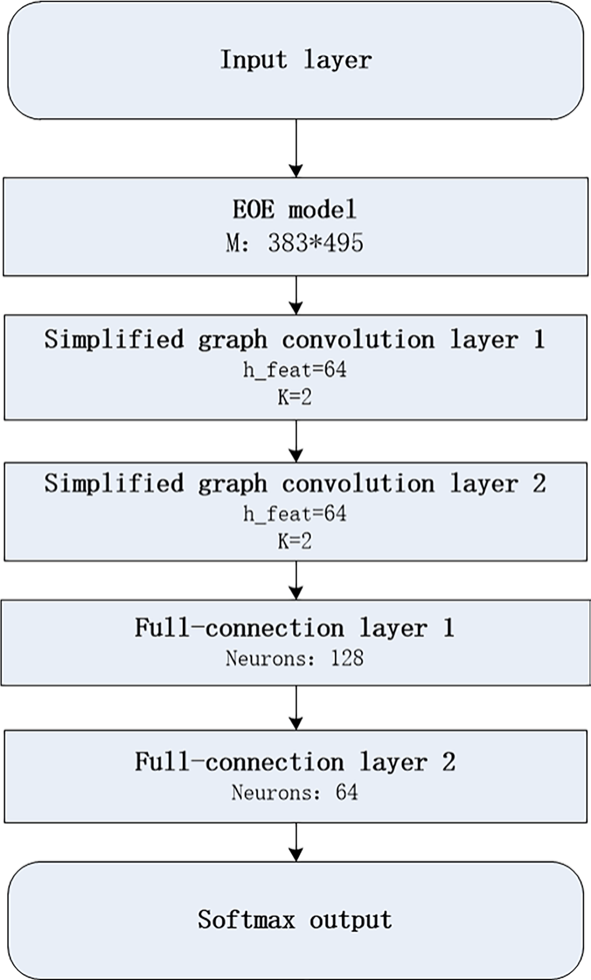
The structure of simplified graph convolutional neural network model is proposed. The input is a vector, and the output is the predicted score for each sample

We combine EOE and SGC models to learn the embedding of nodes, and the two models are trained separately. The main purpose of the EOE model is to add edge information from the coupled heterogeneous graph to nodes, with the similarity matrix of miRNA and disease as the original feature input. The model mainly relies on the loss function to train the feature matrix of miRNA, the feature matrix of disease, and the harmony matrix M. For the construction of graph convolutional network, we adopt two-layer simplified graph convolutional layer construction, each layer gathers two-hop neighbor information, namely K = 2, and the output dimension is 64. MLP consists of two fully connected layers, of which the first layer contains 64 neurons. The details are shown in Fig. 3.

### Experimental approaches and evaluation criteria

**Fig. 4:**
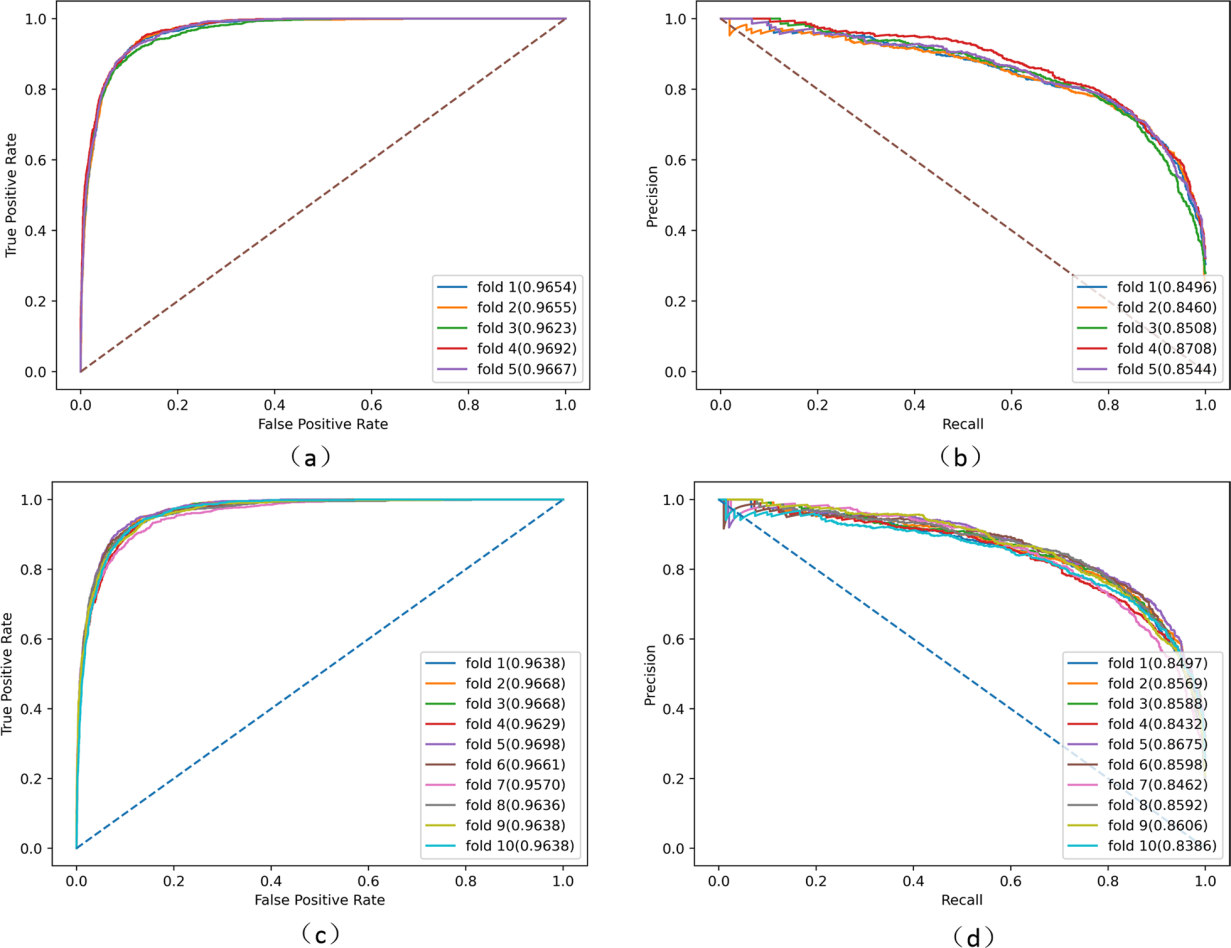
Cross validation results. **a** 5-fold cross-validated ROC curve with a mean AUC of 0.9658; **b** 5-fold cross-validated PR curve with a mean AUPR of 0.8543; **c** 10-fold cross-validated ROC curve with a mean AUC of 0.9644; **d** 10-fold cross-validated PR curve with a mean AUPR of 0.8540

To verify the validity of our proposed EOESGC model, we conduct experiments on the HMDD2.0 database and evaluate the model performance by using 5-fold cross-validation and 10-fold cross-validation. Considering the large difference in the number of positive and negative samples during the experiment, we randomly select 5 negative samples for each positive sample to form the experimental data, thus achieving the function of balancing the data set. The results are shown in Fig. 4. The AUC of our model for 5-fold cross-validation is 0.9658 and the AUPR is 0.8543, the AUC for 10-fold cross-validation is 0.9644 and the AUPR is 0.8540.

### Comparisons with the state-of-the-art methods

To prove the superiority of the proposed model, we compare it with several more excellent models recently proposed, which were LWPCMF [39],   VAGMF [19],   SMALF [18],   CEMDA [40] ,and ICFMDA [41]. The average AUC of the 5-fold cross-validation is used as the evaluation index, and the results are shown in Table 1. Among them, the SMALF model has a better effect, which uses a stacked auto-encoder to learn node features and achieves a better effect, with an AUC value of 0.9505. However, the effect of the EOESGC framework proposed by us is more outstanding, with an AUC value of 0.9658, 1.5$\%$ higher than that of SMALF.

**Table 1:** The AUC of EOESGC and baseline

Method	AUC
EOESGC	0.9658
LWPCMF	0.9411
VAFMF	0.9280
SMALF	0.9503
CEMDA	0.9203
ICFMDA	0.9045

### Parameter sensitivity analysis

**Fig. 5:**
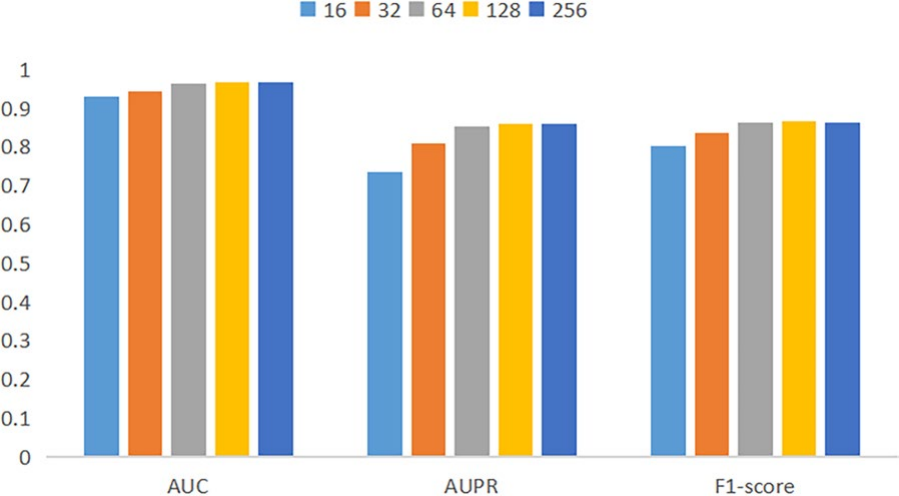
Parameter analysis of EOESGC

Different embedding dimensions will lead to different model training speeds and costs. To select the optimal embedding dimension, we conduct 5-fold cross-validation experiments with different dimensions. The experimental results are shown in Fig. 5. When the embedding dimension is less than 64, the AUC, AUPR, F1-score value shows an upward trend; when the embedding dimension is greater than 64, the evaluation indexes tend to be stable, but the training speed decreases significantly. Therefore, 64 is selected as the feature dimension of the node after comprehensive consideration.

### Compare the different combination types

To verify the effectiveness of learning node embedding in the EOESGC combined model, we conducted an ablation experiment. There are two different kinds of experiments. Category 1 to verify the effectiveness of using the EOE model, we compared this step with the model of a simplified graph convolutional neural network. Category 2 is to verify the effectiveness of the combination of EOE and SGC embedded models. We also select the combination of the other three commonly used graph convolutional neural networks with EOE, namely GCN [35], TAG [42] ,and GraphSage [43]. As shown in Table 2, if edge information is not added as supplementary information for node embedding, the effect of SGC is poor. In addition, the EOE model has a poor combination effect with other commonly used convolution models. Therefore, the experimental results fully prove the validity of this framework.

**Table 2:** The different combination types result

Model	AUC	AUPR	F1-score
EOESGC	0.9658	0.8543	0.8644
SGC	0.9482	0.8134	0.8427
EOEGCN	0.9178	0.7193	0.7973
EOEGraphSAGE	0.9301	0.7685	0.8169
EOETAG	0.9501	0.8147	0.8419

### Case study

Breast neoplasms are common cancers that threaten women’s health worldwide and are also one of the leading causes of nausea in women’s deaths [44]. In recent years, gene diagnosis and gene therapy of breast cancer has become a hot topic. Studies have shown that miRNA, as a regulatory factor, plays an important role. For example, low expression of mir-195 can be easily observed in breast cancer cell lines and tissue samples from chemotherapy-sensitive or drug-resistant patients [44]. In addition, mir-195 can decrease the survival rate and increase apoptosis of breast tumor cells by down-regulating the expression of Raf-1, Bcl-2 ,and P-glycoprotein [44]. Therefore, it is necessary to use advanced methods to predict the potential miRNA related to breast neoplasms, so we predict the top 20 miRNAs related to breast tumors, as shown in Table 3. All the miRNAs we predict can be found in the validation database.

**Table 3:** The top 20 potential miRNAs related to Breast Neoplasms

miRNA	Evidence	miRNA	Evidence
hsa-mir-142	dbDEMC,HMDD3.2	hsa-mir-106a	dbDEMC,HMDD3.2
hsa-mir-150	dbDEMC,HMDD3.2	hsa-mir-574	dbDEMC,HMDD3.2
hsa-mir-181c	dbDEMC,HMDD3.2	hsa-mir-15b	dbDEMC,HMDD3.2
hsa-mir-192	dbDEMC,HMDD3.2	hsa-mir-30e	dbDEMC,HMDD3.2
hsa-mir-494	dbDEMC,HMDD3.2	hsa-mir-138	dbDEMC,HMDD3.2
hsa-mir-378a	dbDEMC,HMDD3.2	hsa-mir-424	dbDEMC,HMDD3.2
hsa-mir-184	dbDEMC,HMDD3.2	hsa-mir-372	dbDEMC,HMDD3.2
hsa-mir-208b	dbDEMC	hsa-mir-212	dbDEMC,HMDD3.2
hsa-mir-208a	dbDEMC,HMDD3.2	hsa-mir-134	dbDEMC,HMDD3.2
hsa-mir-99a	dbDEMC,HMDD3.2	hsa-mir-28	dbDEMC

Lung neoplasms are the most common type of nausea and have a high mortality rate. Previous studies have shown that miRNA is involved in almost every process of lung cancer, including tumor progression, angiogenesis, invasion ,and metastasis. For example, the expression level of miR-29s was found to be inversely correlated with DNA methyltransferase 3A (DNMT3A) and DNA methyltransferase 3B (DNMT3B) in lung cancer tissues by controlling methylation to inhibit the reexpression of tumor suppressor genes and inhibit tumorigenesis [45]. The first 20 miRNAs associated with lung cancer were predicted using our proposed framework, as shown in Table 4, among which the first 19 miRNAs are successfully verified.

**Table 4:** The top 20 potential miRNAs related to Lung Neoplasms

miRNA	Evidence	miRNA	Evidence
hsa-mir-16	dbDEMC,HMDD3.2	hsa-mir-378a	dbDEMC
hsa-mir-122	dbDEMC,HMDD3.2	hsa-mir-20b	dbDEMC
hsa-mir-15a	dbDEMC,HMDD3.2	hsa-mir-23b	dbDEMC
hsa-mir-106b	dbDEMC,HMDD3.2	hsa-mir-184	dbDEMC
hsa-mir-195	dbDEMC,HMDD3.2	hsa-mir-342	dbDEMC,HMDD3.2
hsa-mir-429	dbDEMC	hsa-mir-208a	HMDD3.2
hsa-mir-373	dbDEMC,HMDD3.2	hsa-mir-99a	dbDEMC,HMDD3.2
hsa-mir-451a	dbDEMC,HMDD3.2	hsa-mir-302b	dbDEMC
hsa-mir-141	dbDEMC,HMDD3.2	hsa-mir-15b	dbDEMC
hsa-mir-302a	dbDEMC	hsa-mir-208b	Unconfirmed

## Conclusions

Experiments show that our proposed EOESGC framework can effectively predict the potential miRNA-disease associations. In the coupled heterogeneous graph, EOE is used to add edge information to node embedding, which makes node embedding contain richer and more comprehensive information. Then the SGC model is used to aggregate the node information. Finally, the results are predicted using MLP. We combine EOE and SGC models for the first time. The two models play different roles respectively, but their purpose is to learn the effective feature embedding of nodes. To simplify the computational complexity and ensure the edge validity in the coupled heterogeneous graph, we simplify the graph structure twice. The AUC value of EOESGC model based on 5-fold cross-validation is 0.9650, which is higher than that of previous methods. The top 20 associated potential miRNAs are predicted in lung and breast cancer cases.dbDEMC and HMDD3.2 databases are used in the validation database, and 20, 19 miRNAs are identified in the validation database. Therefore, the EOESGC framework is very effective for predicting the potential miRNA-disease associations.

Although our proposed framework can effectively predict the miRNA-disease potential association, we cannot predict the miRNAs associated with new diseases. If the original data does not contain the known miRNAs of the disease, we cannot predict the unknown miRNAs. Therefore, in the next step, we need to solve the problem of how to effectively predict the potential miRNAs of new diseases.

## Data Availability

The datasets used and/or analysed during the study is available from the corresponding author on reasonable request. Data can be downloaded from the Human miRNA Disease Database: http://www.cuilab.cn/hmdd/.
